# Limited Sensitivity of Hippocampal Synaptic Function or Network Oscillations to Unmodulated Kilohertz Electric Fields

**DOI:** 10.1523/ENEURO.0368-20.2020

**Published:** 2020-12-17

**Authors:** Zeinab Esmaeilpour, Mark Jackson, Greg Kronberg, Tianhe Zhang, Rosana Esteller, Brad Hershey, Marom Bikson

**Affiliations:** 1 Neural Engineering Laboratory, Department of Biomedical Engineering, The City College of the City University of New York, City College Center for Discovery and Innovation, New York, 10031 NY; 2 Boston Scientific Neuromodulation Research and Advanced Concepts, 25155 Rye Canyon Loop, Valencia, CA 91355

**Keywords:** brain stimulation, γ oscillation, high-frequency stimulation, kilohertz electrical stimulation, neuronal excitability

## Abstract

Understanding the cellular mechanisms of kilohertz (kHz) electrical stimulation is of broad interest in neuromodulation including forms of transcranial electrical stimulation, interferential stimulation, and high-rate spinal cord stimulation (SCS). Yet, the well-established low-pass filtering by neuronal membranes suggests minimal neuronal polarization in respond to charge-balanced kHz stimulation. The hippocampal brain slice model is among the most studied systems in neuroscience and exhaustively characterized in screening the effects of electrical stimulation. High-frequency electric fields of varied amplitudes (1–150 V/m), waveforms (sinusoidal, symmetrical pule, asymmetrical pulse) and frequencies (1 and10 kHz) were tested. Changes in single or paired-pulse field EPSPs (fEPSP) in CA1 were measured in response to radial-directed and tangential-directed electric fields, with brief (30 s) or long (30 min) application times. The effects of kHz stimulation on ongoing endogenous network activity were tested in carbachol-induced γ oscillation of CA3a and CA3c. Across 23 conditions evaluated, no significant changes in fEPSP were resolved, while responses were detected for within-slice control direct current (DC) fields; 1-kHz sinusoidal and pulse stimulation (≥60 V/m), but not 10 kHz, induced changes in oscillating neuronal network. We thus report no responses to low-amplitude 1-kHz or any 10-kHz fields, suggesting that any brain sensitivity to these fields is via yet to be-determined mechanism(s) of action which were not identified in our experimental preparation.

## Significance Statement

There a large mismatch between enthusiasm for clinical treatments using kilohertz (kHz) frequency electrical stimulation and the understanding of kHz mechanisms of action. Indeed, the well-established low-pass properties of cell membranes should attenuate any response to kHz stimulation. This study presents the largest and broadest characterization of the cellular effects of kHz stimulation using the most established animal model to detect CNS sensitivity to electric fields: our work systematically evaluated sensitivity of hippocampal synaptic function and oscillatory network activity in response to kHz. Only at low kHz (1 but not 10 kHz) with high intensity and during oscillations, responses were detected. This systematic and largely negative experimental series suggest kHz neuromodulation operates via yet to be determined mechanisms.

## Introduction

Electric fields at low frequencies (<100 Hz) are highly effective in changing firing rate and timing of neuronal population (McIntyre et al., 2004; Fröhlich and McCormick, 2010), including at very low (∼1 V/m) intensities (Reato et al., 2010). However, as the frequency of electric field oscillations increases beyond a few hundred hertz, sensitivity to stimulation and brain responses diminishes (Deans et al., 2007). On the one hand, this is readily attributable to the low-pass filtering characteristics of cell membranes (Ranck, 1975; McIntyre and Grill, 1999; Bikson et al., 2004; Deans et al., 2007). Emerging neuromodulation techniques specifically using kHz frequency stimulation have been developed, in some cases with marked clinical efficacy. This includes transcranial alternating current stimulation (tACS) with sinusoidal kHz waveforms (Chaieb et al., 2011), transcranial random noise stimulation (tRNS; Terney et al., 2008; Antal and Paulus, 2013; Laczó et al., 2014), kHz spinal cord stimulation (SCS; Kapural et al., 2015; De Carolis et al., 2017), and, recently, kHz deep brain stimulation (DBS; Harmsen et al., 2019; Khadka et al., 2020). Approaches using interferential or intersectional short pulse stimulation (Grossman et al., 2017; Vöröslakos et al., 2018; Esmaeilpour et al., 2020) are a special case underpinned by an assumption of sensitivity to amplitude-modulated (AM) kHz field, but no responses to unmodulated kHz stimulation.

Across this proliferation of techniques and application of kHz neuromodulation, the cellular mechanisms of kHz electrical stimulation remain unclear (Dmochowski and Bikson, 2017; Pelot et al., 2017). While, at very high stimulation intensities, kHz stimulation may produce supraphysiological changes [e.g., conduction block (Zhang et al., 2006; Crosby et al., 2017), electroporation (Dowden et al., 2010)], for existing clinical applications these intensities are not expected at target tissue. Given that the response of neurons to kHz electrical stimulation is attenuated, the possibility of subthreshold stimulation of baseline neuronal activity (where ongoing neuronal activity is modulated; Bikson et al., 2013b) is considered alongside supra-threshold stimulation (*de novo* generation of action potentials/pacing).

Our goal was to systematically evaluate the sensitivity of hippocampal synaptic function and oscillatory network activity to kilohertz (kHz) frequency extracellular electrical stimulation. For assessing the sub and supra-threshold effects of electric stimulation on brain excitability, the application of uniform electric fields across the rodent slice preparation is among the longest-standing and most exhaustively studied animal models (Jefferys, 1981; Bikson et al., 2004; Jackson et al., 2016). fEPSPs, including pair-pulse responses, are sensitive to modulation by electric fields through changes in axonal excitability (Kabakov et al., 2012; Rahman et al., 2017), synaptic activity (Rahman et al., 2013), dendritic activity (Bikson et al., 2004; Kronberg et al., 2017), and somatic activity (Radman et al., 2009; Fröhlich and McCormick, 2010), while generally providing a global index for excitatory and inhibitory synaptic efficacy (Jefferys, 1981), information processing (Gluckman et al., 1996; Radman et al., 2007; Lafon et al., 2017), and plasticity (Fritsch et al., 2010; Ranieri et al., 2012; Kronberg et al., 2017). Neuronal network oscillations, including those in the γ frequency band, are highly sensitive to electric fields through well-characterized mechanisms of amplification (Deans et al., 2007; Fröhlich and McCormick, 2010; Reato et al., 2010).

Here, we use fEPSP and oscillations to test the effect of 1- and 10-kHz electrical stimulation using sinusoidal symmetric and asymmetric pulse waveforms. We used direct current (DC) electrical stimulation as a within-slice control to confirm the sensitivity to low-frequency stimulation. Our data suggest the presence of diminished neuronal sensitivity in response to kHz stimulation consistent with the dramatic low-pass filtering property of the neuronal membrane. Oscillatory networks (e.g., γ oscillation) are more sensitive to electrical stimulation but only to 1-kHz stimulation at ≥60-V/m intensity. Thus, consistent with results using sub-kHz electric fields, the structure of ongoing network oscillations would determine maximal sensitivity and effects of stimulation (Reato et al., 2013). If the brain is sensitive to high-kHz frequencies (i.e., 10 kHz) or lower-amplitude stimulation, it may be via mechanisms yet to be identified in the brain slice preparation (e.g., peculiarly sensitive neuronal elements, non-neuronal elements such as neuroglia, vascular response, heating), effects peculiar to non-uniform fields, and/or effects with a gradual (e.g., hours) onset.

## Materials and Methods

All animal experiments were conducted in accordance with guidelines and protocols approved by the Institutional Animal Care and Use Committee at The City Collage of New York (CUNY).

### Hippocampal slice preparation

Hippocampal brain slices were prepared from male Wistar rats aged three to five weeks old, which were deeply anaesthetized with ketamine (7.4 mg kg^−1^) and xylazine (0.7 mg kg^−1^) applied intraperitoneally and killed by cervical dislocation. The brain was quickly removed and immersed in chilled (2–6°C) dissecting solution containing the following: 110 mm choline chloride, 3.2 mm KCl, 1.25 mm NaH_2_PO_4_, 26 mm NaHCO_3_, 0.5 mm CaCl_2_, 7 mm MgCl_2_, 2 mm sodium ascorbate, 3 mm sodium pyruvate, and 10 mm D-glucose. Transverse hippocampal slices (400 μm thick) were cut using a vibrating microtome (Campden Instruments) and transferred to a recovery chamber for 30 min at 34°C with a modified artificial CSF (ACSF) containing the following: 124 mm NaCl, 3.2 mm KCl, 1.25 mm NaH_2_PO_4_, 26 mm NaHCO_3_, 2.5 mm CaCl_2_, 1.3 mm MgCl_2_, 2 mm sodium ascorbate, 3 mm sodium pyruvate, and 25 mm D-glucose. Slices were then transferred to a holding chamber for at least 30 min (or until needed) at 30°C with ACSF containing the following: 124 mm NaCl, 3.2 mm KCl, 1.25 mm NaH_2_PO_4_, 26 mm NaHCO_3_, 2.5 mm CaCl_2_, 1.3 mm MgCl_2_, and 25 mm D-glucose. For fEPSP experimental recordings, slices were then transferred to a fluid–gas interface recording chamber (Hass top model, Harvard Apparatus) perfused with warmed ACSF (30.0 ± 0.1°C) at 1.0 ml min^−1^. For γ oscillation experiments, slices were transferred to a fluid–gas interface recording at 34°C. All solutions were saturated with a gas mixture of 95% O_2_–5% CO_2_. γ Oscillations were induced by perfusing the slices with ACSF containing 20 μm carbachol (carbamoylcholine chloride). All reagents were purchased from Sigma-Aldrich.

### fEPSP recording (acute and long term)

**Figure 1.**
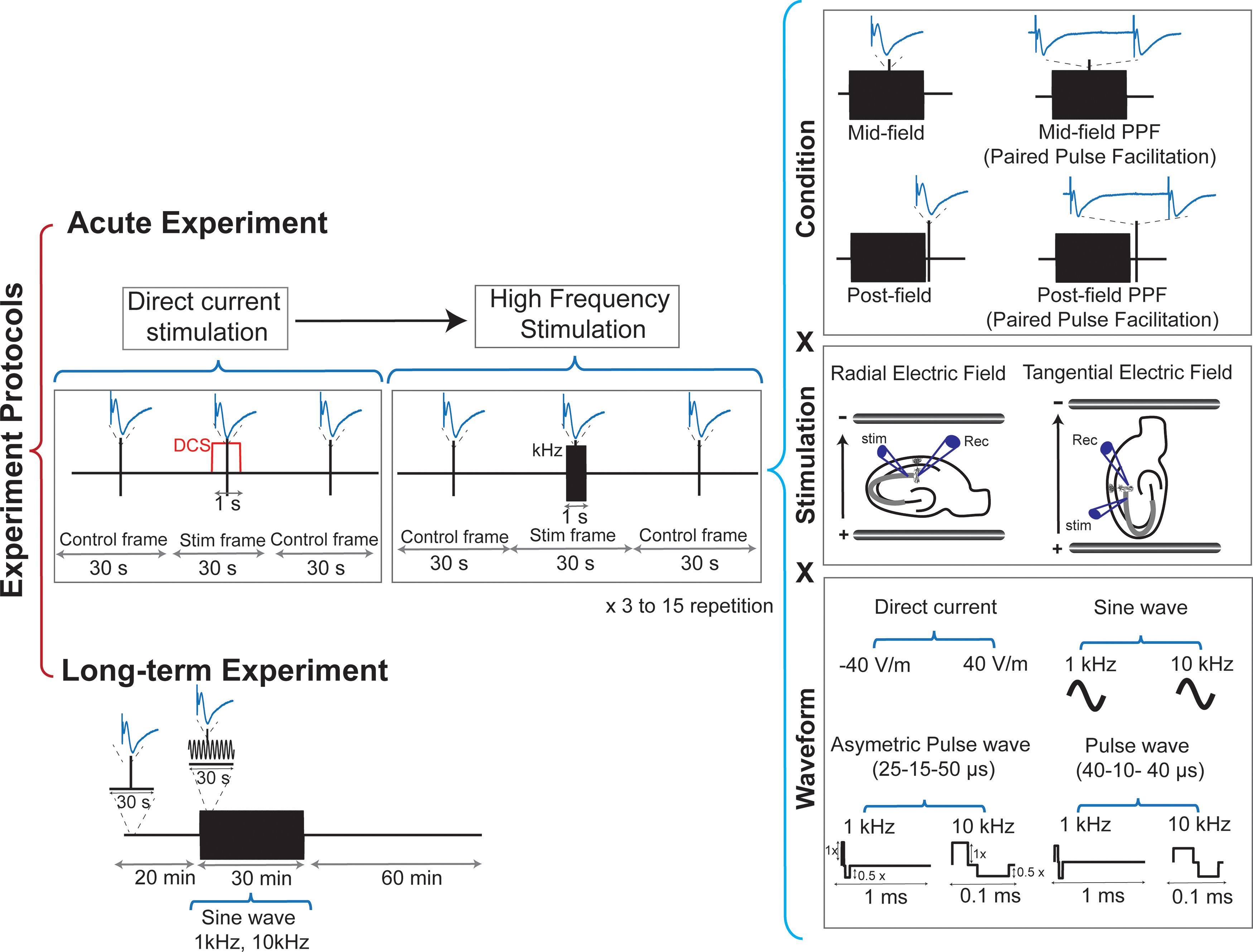
Experimental design of hippocampal slice recordings. Acute experiments: DC stimulation as within-slice control condition before high-frequency stimulation paradigm. fEPSP was evoked and recorded in four different conditions: MF, mid-filed PPF, PF, and PF PPF. Bipolar stimulation and glass recording electrodes depicted in CA1 stratum radiatum along with a pyramidal neuron and Schaffer collateral (gray). Stimulation: field wires were placed on opposite sides across the slice and connected to a current source. In radial configuration electric fields were applied parallel to the CA1 pyramidal somato-dendritic axis and in tangential configuration, electric fields were applied perpendicular to the CA1 pyramidal somato-dendritic axis. Waveform: DC and various electric field waveforms for kHz stimulation. The duration of each waveform component is given in μs for 1 kHz and 10-kHz stimulation. Alternating control and kHz (or DC) epochs were repeated every 30 s. Raw data were low pass filtered to obtain fEPSPs for analysis. fEPSP obtained during kHz/DCS (MF) or 0.1 ms after kHz/DCS (PF) were normalized to the average of proceeding and following fEPSP. Long-term experiment: fEPSP was evoked every 30 s. Stimulation was applied for 30 min after a 20-min stable baseline. fEPSP recording was continued 1 h after the end of stimulation.

Recordings started 30 min after transfer to the recording chamber. fEPSPs were evoked in the Schaffer collateral pathway using a platinum–iridium bipolar stimulating electrode placed in stratum radiatum of CA1 ∼300 μm from stratum pyramidale. Recording electrodes made from glass micropipettes (aluminosilicate glass with 1.5-mm outer diameter, 1.0-mm inner diameter) pulled by a Sutter Instruments P-97 and filled with ACSF (resistance 0.5–2 MΩ) were placed in stratum radiatum of CA1, ∼400 μm from the stimulating electrode and within 100 μm from stratum pyramidale (Fig. 1). fEPSPs were quantified by the average initial slope, taken during the first 0.5 ms after the onset of the fEPSP. Stimulus intensity was set to evoke fEPSPs with 35–50% of the maximum slope, which was determined at the onset of recording. For paired-pulse facilitation (PPF) experiments, two fEPSPs were evoked at a 50-ms interval (Korte et al., 1995; Lessmann and Heumann, 1998; Kronberg et al., 2017). PPF was quantified as the ratio of the second to the first fEPSP slope in each condition.

For acute experiments, fEPSPs were evoked every 30 s, alternating between control and kHz (or Direct current stimulation (DCS)) conditions. Waveforms were applied for 1 s and fEPSPs were evoked midway (0.5 s, mid-field; MF) through the stimulation (Fig. 1). Where indicated, fEPSPs were also evoked 0.1 ms after the extracellular field was turned off (post-field; PF). For control conditions, fEPSPs were evoked alone (no kHz stimulation). Within a given slice, a single kHz waveform was tested at multiple intensities in a randomized order ranging from 1 to 80 V/m (1, 5, 10, 20, 40, 60, and 80 V/m) with each intensity repeated 3–15 times per slice. fEPSP slopes during each kHz epoch were normalized to the average of the control fEPSP slopes immediately preceding and following it. Normalized fEPSP slopes were then averaged across the repeats for each intensity, producing one *n* per slice per waveform.

For long-term experiments, fEPSPs were evoked every 30 s and fEPSP slope was monitored online. After at least 30 min of stable baseline fEPSP recordings, 1 and 10 kHz waveforms were applied parallel to the somato-dendritic axis (radial) at 80 V/m for 30 min. fEPSPs were continuously evoked every 30 s throughout the kHz and for 60 min after kHz ended. To determine stability before stimulation, a least squares linear fit was applied to the baseline fEPSP slopes. The slope of the linear fit (mV ms^−1^ min^−1^) was required to be <0.33% of the mean baseline fEPSP slopes (i.e., <20% drift expected over 60 min). For the control condition, the same stability criteria were used, but no stimulation was applied. To quantify long-term effects, fEPSP slopes were normalized to the mean of the 20 min immediately preceding high-frequency stimulation. Sampling frequency was reduced to 10 kHz during long-term experiments in both 1- and 10-kHz stimulation because of technical limitations. The responses were compared between sham and control condition in three different times (immediately, 30 min, and 60 min after termination of stimulation).

### Data analysis

All data are reported as the mean ± SEM. Reported n values represent the number of slices used in each condition. Statistical analysis was performed using unpaired, one sample *t* test for positive and negative DC control stimulation, after checking for normality in each group (Lilliefors test for normality, *p* > 0.05 in all cases) and one-way repeated measure ANOVA for different intensities used in kHz waveforms. Bonferroni correction was used for multiple comparison correction. All the analysis was performed in R (RStudio).

### Bayesian inference

Difference across highest electric field intensity and baseline were analyzed using the Bayesian paired samples *t* test as implemented in JASP v0.13.1.0 using default effect size prior (Cauchy 0.707; Keysers et al., 2020). Results are reported using two tailed Bayes factor BF_+0_ that represents p(H_+_|80 v/m ≠ baseline)/p(H_0_|80 v/m = baseline). Effect size estimates are reported as median posterior Cohen’s *δ* with 95% credibility interval (CI) using a two-tailed H_1_ in order not to bias estimates in the expected direction. Bayesian ANOVAs were conducted using JASP with default priors, and effects are reported as Bayes factor for the inclusion of a particular effect, calculated as the ratio between the likelihood of the data given the model with versus the next simpler model without that effect.

### Electrical filed stimulation

kHz and DCS extracellular electric fields were applied to slices via two parallel Ag–AgCl wires (1-mm diameter, 12-mm length, 10 mm apart) placed in the recording chamber on opposite sides of the brain slice with the recording site approximately equidistant from each wire. Slices were oriented so that the resulting electric field was either parallel (radial stimulation) or perpendicular (tangential stimulation) to the somato-dendritic axis of CA1 pyramidal neurons (Fig. 1). In CA3 experiments, slices were oriented so that the resulting electric field was parallel to the main somato-dendritic axis of CA3a pyramidal neurons (perpendicular to pyramidal cell layer; Fig. 1*A.1*). Field wires were connected to a custom high band-width voltage-controlled isolated current source. Before each recording, the applied current intensity was calibrated by measuring the electric field (voltage difference between two recording electrodes separated by 0.8 mm in the slice) in response to a 10-μA DC test pulse. This characterized the linear relationship between electric field magnitude and applied current, which was then used to determine the current intensity required for a desired electric field. Data acquisition and stimulation waveforms were controlled by Power1401–625 kHz hardware and Signal software version 6.0 [Cambridge Electronic Design (CED)]. Voltage signals were amplified (10×), analog low pass filtered (20 kHz; Model 3000 differential amplifier, A-M Systems) and digitized (200 kHz, Power1401–625 kHz and Signal, CED). Before analyzing the fEPSP slope, all signals were digitally low pass filtered with Signal 6.0 (FIR filter, 2047 coefficients, 250-Hz transition gap, 1099 −3 dB) or MATLAB to remove stimulation artifact (700-Hz cutoff for 1-kHz stimulation and 1-kHz cutoff for 10-kHz stimulation).

kHz was applied at 1 and 10 kHz using the following kHz waveforms (leading polarity pulse width, interphase interval, opposite polarity pulse width): sinusoid, pulse (40–10–40 μs for 1 and 10 kHz), and an asymmetric pulse waveform with the shorter duration pulse at 2× the amplitude of the longer duration pulse (25–15–50 μs for 10 kHz; Fig. 1). Reported magnitude for the asymmetric pulse waveform is the electric field during the leading (shorter) pulse. For each slice, DCS at 40 V/m was applied with alternating polarity before kHz waveforms as a basis for comparing effect sizes. Here, positive, radial +DCS refers to uniform DC electric fields that are parallel to the somato-dendritic axis of CA1 pyramidal neurons, with the positive terminal closer to the apical dendrites (as opposed to basal dendrites). Positive, tangential DCS refers to uniform DC electric fields that are parallel to Schaffer collaterals in CA1 with DCS current flow in the same direction as orthodromic action potential propagation (Fig. 1). Unless otherwise stated, the electric field reported throughout the manuscript is the peak electric field for each waveform.

### Extracellular recordings (γ oscillation)

Recordings of extracellular field potentials in the pyramidal layer of CA3a and CA3c region of hippocampus were obtained using glass micropipettes (15 MΩ pulled on a P-97, Sutter Instruments) field with ACSF. Data acquisition and electrical stimulation were controlled by Power1401–625 kHz hardware and Signal software version 6.0 (CED). Voltage signals were amplified (10×), analog low pass filtered (20 kHz; Model 3000 differential amplifier, A-M Systems) and digitized (20 kHz, Power1401–625 kHz and Signal, CED). To reduce noise and stimulation artifacts, the voltage recordings were always performed relative to an iso-potential electrode placed in bath (Fig. 5*A.1*). Field recordings overcome potential limitations of intracellular recording during kHz field such as current collection by the capacitive-walled microelectrode leading to artifactual intracellular stimulation (FallahRad et al., 2019) or possible amplifier distortion (Lesperance et al., 2018).

### Power analysis and statistics

Signals were recorded in frames of 5 s (1.5 s before and 1.5 s after stimulation) and stimulation was applied for 2 s. Stimulation artifacts were minimized by subtracting the voltage in an iso-potential refence electrode from the recording electrode in the slice (Fig. 5). Spectrograms were computed (200-ms hamming window, 90% overlap) on individual 5-s frames and averaged over 100 frames for each stimulation condition (i.e., frequency, waveform and amplitude). Normalized power was measured as a power ratio normalized by prestimulation power in the frequency band of the endogenous oscillation. Mean γ power was calculated in the center frequency of oscillation (5-Hz window). To quantify the slope of poststimulation, a line was fitted within a 300-ms window immediately after stimulation turned off using the “polyfit” function in MATLAB 2016b (MathWorks Inc). All the results are reported as mean ± SEM; *n* = number of slices. For statistical analysis paired *t* test was used to compare poststimulation and prestimulation in each electric field intensity and significance level (*p*) was corrected using Bonferroni for multiple (e.g., for four comparisons made in each experiment, *p* < 0.0125 was considered significant). All the analysis was performed in R (RStudio).

## Results

### Effect of kHz stimulation on hippocampal field potentials in CA1

Field EPSPs (fEPSPs) measured at dendrites reflect the aggregate postsynaptic current entering to a population of neurons, which is a measure of synaptic input. fEPSPs are sensitive to low-frequency electric fields (Bikson et al., 2004; Lafon et al., 2017). Using rat hippocampal slice preparation, we tested the acute and long-term effects of uniform unmodulated kHz electric fields on synaptic efficacy with electric field direction in parallel or perpendicular to primary somato-dendritic axis (Bikson et al., 2004). The effects of DC electric field were also assessed as within-slice positive controls. fEPSPs were evoked in CA1 region of rat hippocampus by activating the Schaffer collateral pathway. Unless otherwise stated, changes in fEPSP slope from electric field application were calculated as a ratio of slope during electric field application versus control (i.e., no stimulation). PPF which is a measure of short-term synaptic plasticity was used in our recording and was calculated as the ratio of the second fEPSP slope to the first (50-ms interpulse interval) in each condition. Unless otherwise stated, results are reported as mean ± SEM and stimulation were applied for 1 s in all acute experiments and 30 min in long-term experiments.

**Figure 2.**
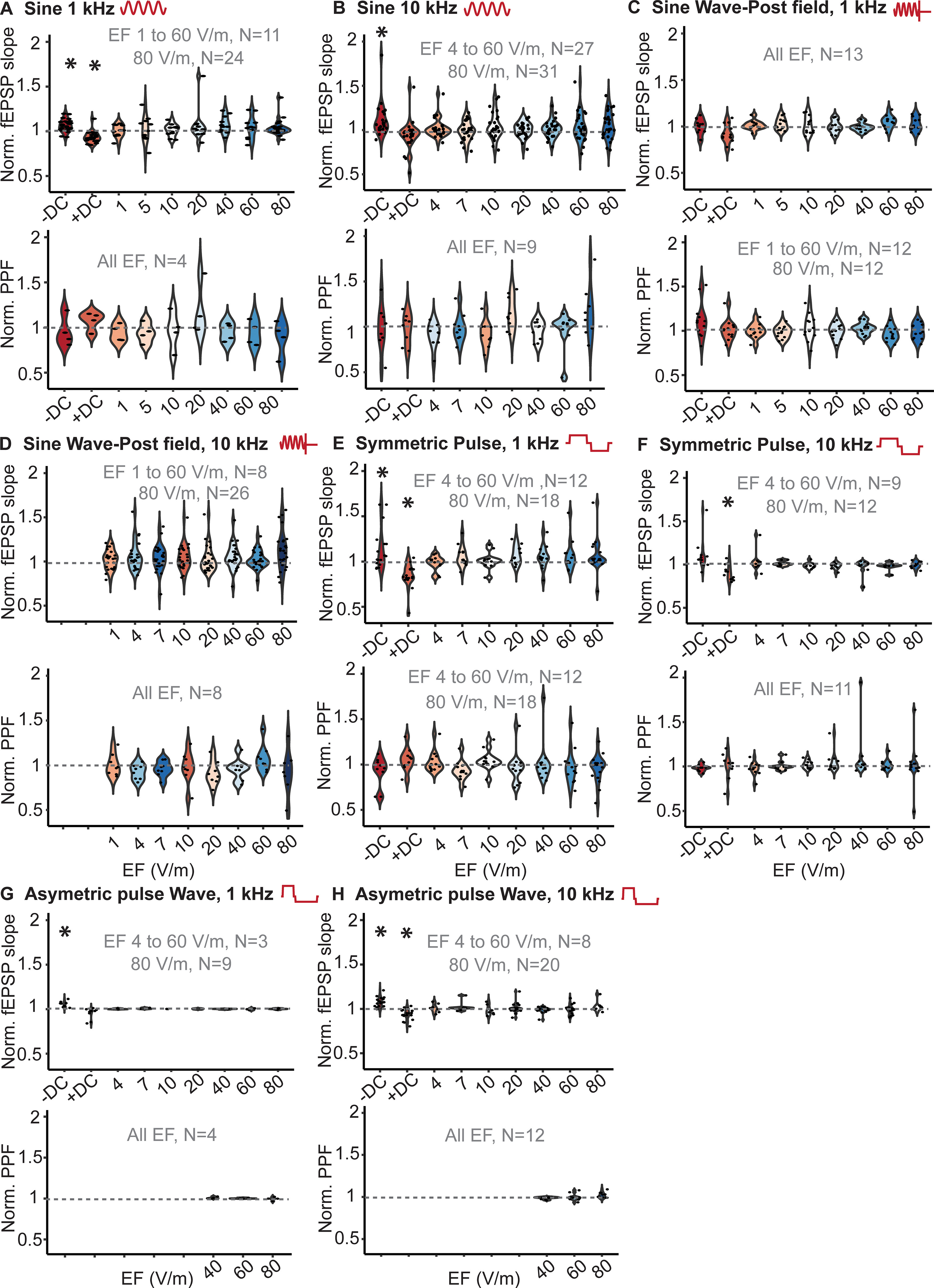
Acute effect of DC and high-frequency electrical stimulation in radial electric field. ***A***, Normalized slope of fEPSP and PPF during positive and negative 40 V/m DC and 1-kHz sinusoidal stimulation. ***B***, Normalized slope of fEPSP and PPF during positive and negative 40 V/m DC and 10-kHz sinusoidal stimulation. ***C***, Normalized slope of fEPSP and PPF immediately after 1-kHz sinusoidal stimulation (PF). ***D***, Normalized slope of fEPSP and PPF immediately after positive and negative 40 V/m DC and 10-kHz sinusoidal stimulation (PF). ***E***, Normalized slope of fEPSP and PPF during positive and negative 40 V/m DC and 1-kHz symmetric pulse waveform stimulation. ***F***, Normalized slope of fEPSP and PPF during positive and negative 40 V/m DC and 10-kHz symmetric pulse waveform stimulation. ***G***, Normalized slope of fEPSP and PPF during positive and negative 40 V/m DC and 1-kHz asymmetric pulse waveform stimulation. ***H***, Normalized slope of fEPSP and PPF during positive and negative 40 V/m DC and 10-kHz asymmetric pulse waveform stimulation. Black circles indicate each data point. Recording frame was 30 s long in all the acute experiments. Stimulation was applied for 1 s in the middle of the recording frame (14.5 −15.5 s). Each data point represents average of 3–15 repetition. *N*, the number of hippocampal slices in each intensity; EF, electric field; *p < 0.05.

When electric fields were applied in the radial direction (electric field parallel to the somato-dendritic axis of CA1 pyramidal neurons), sinusoidal stimulation with 1 kHz did not produce significant effects (*F*_(6,75)_ = 0.5835, ns) in any of intensities tested (1, 5, 10, 20, 40, 60, 80 V/m). However, DC stimulation significantly modulated fEPSP slope [–DC (1.06 ± 0.014, *N* = 24, *p* < 0.01) +DC (0.932 ± 0.0127, *N* = 24, *p* < 0.01)]. Neither DC nor 1-kHz sinusoidal stimulation affected PPF. Increasing stimulation frequency from 1 to 10 kHz (fEPSP, 10 kHz: *F*_(6,160)_ = 0.86, ns; PPF, 10 kHz: *F*_(6,55)_ = 2.8, ns), or changing recording time from during stimulation to immediately after the field was turned off (fEPSP, 1 kHz *F*_(6,66)_ = 1.21, ns; PPF *F*_(6,66)_ = 0.88, ns; fEPSP, 10 kHz *F*_(7,175)_ = 2.2, ns, PPF *F*_(7,47)_ = 1.316, ns) did not modulate fEPSP over the range of electric field intensities tested (Fig. 2*B*,*C*).

Symmetric and asymmetric charge-balanced waveforms are ubiquitous in implanted stimulators including DBS and SCS. Stimulation with radially-directed symmetric pulse waveforms at 1- and 10-kHz electric fields did not modulate fEPSP (1 kHz, *F*_(6,73)_ = 0.788, ns; 10 kHz, *F*_(6,50)_ = 1.03, ns) or PPF (1 kHz, *F*_(6,72)_ = 1.30, ns; 10 kHz, *F*_(6,61)_ = 0.68, ns; Fig. 2*E*,*F*). Radially directed electric fields with asymmetric pulse waveform also did not modulate fEPSP or PPF regardless of frequency (fEPSP: 1 kHz, *F*_(6,15)_ = 0.63, ns; 10 kHz, *F*_(6,84)_ = 1.022, ns; PPF: 1 kHz, *F*_(2,9)_ = 0.72, ns; 10 kHz, *F*_(2,32)_ = 0.86, ns; Fig. 2*G*,*H*).

**Figure 3.**
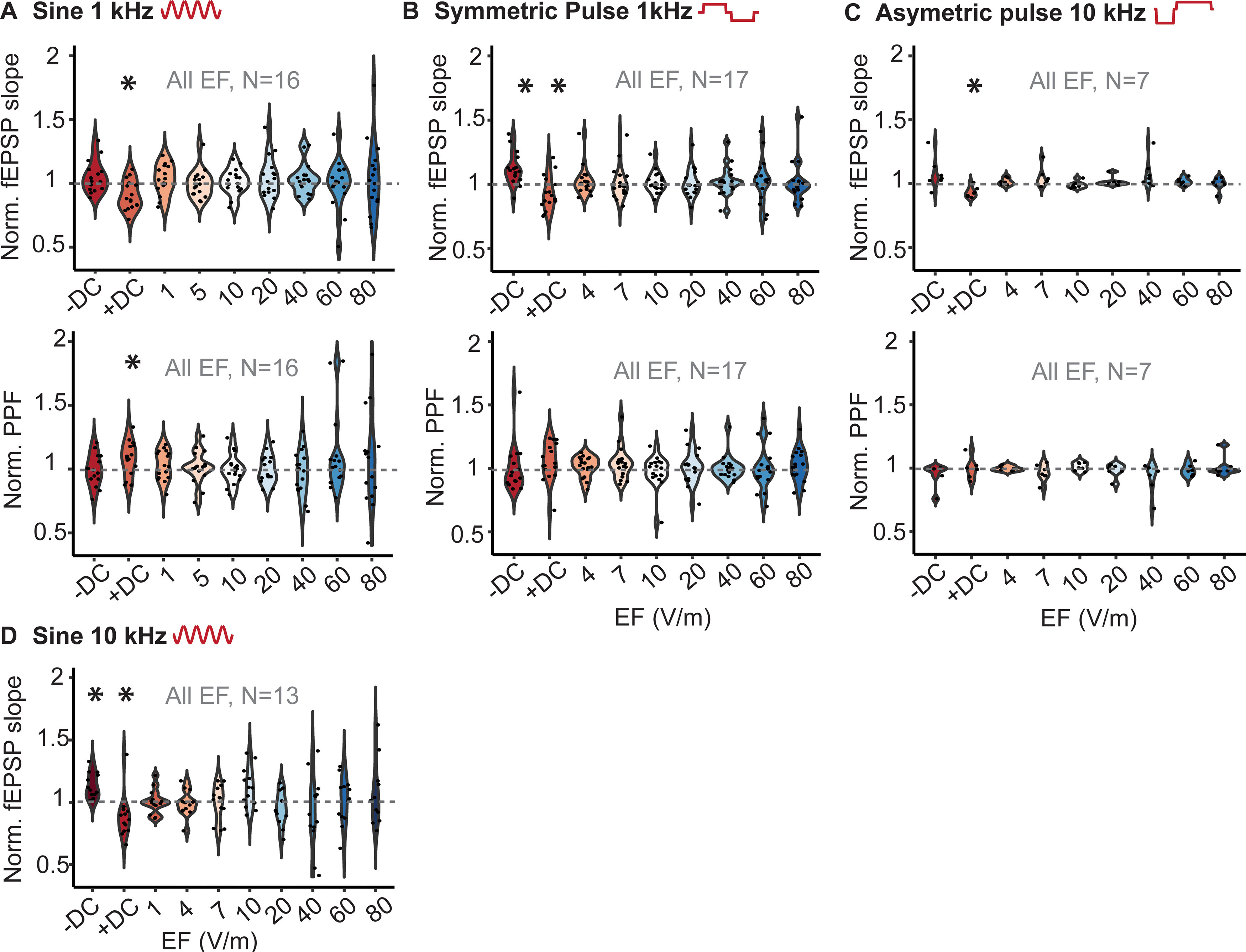
Acute effect of DC and high-frequency stimulation in tangential direction. ***A***, Normalized slope of fEPSP and PPF during positive and negative 40 V/m DC and 1-kHz sinusoidal stimulation. ***B***, Normalized slope of fEPSP and PPF during positive and negative 40 V/m DC and 1-kHz symmetric pulse waveform. ***C***, Normalized slope of fEPSP and PPF during positive and negative 40 V/m DC and 10-kHz asymmetric pulse waveform. ***D***, Normalized slope of fEPSP during positive and negative 40 V/m DC and 10-kHz asymmetric sine waveform. Colored circles indicate different data point. *N*, the number of hippocampal slices; EF, electric field; *p < 0.05.

When electric field was applied in tangential direction (i.e., perpendicular to somato-dendritic axis of CA1 pyramidal neurons), sinusoidal waveform (1 kHz: fEPSP, *F*_(6,105)_ = 0.231, ns, PPF, *F*_(5,90)_ = 0.58, ns; 10 kHz: fEPSP *F*_(7,83)_ = 1.52, ns; Fig. 3*A*,*D*), symmetric (1 kHz: fEPSP, *F*_(6,96)_ = 0.08, ns, PPF, *F*_(6,96)_ = 0.52, ns), and asymmetric waveforms (10 kHz: fEPSP, *F*_(6,36)_ = 1.71, ns, PPF, *F*_(6,41)_ = 1.30, ns), at 1 kHz or 10 kHz, did not modulate fEPSPs.

**Figure 4.**
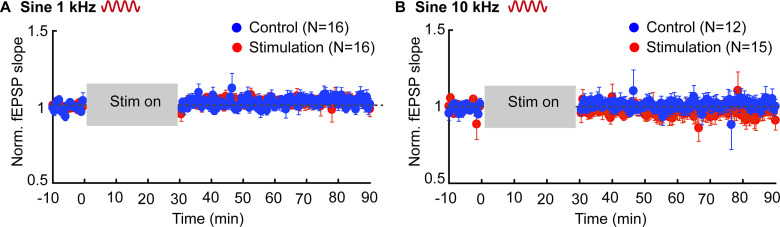
Long-term effect of kHz stimulation on synaptic efficacy. ***A***, Normalized fEPSP slope in response to 30-min stimulation (between 0 and 30) 1-kHz sine waveform, 80 V/m in radial direction after at least 20-min stable baseline. Follow-up recording continued for 60 min after stimulation. ***B***, Normalized fEPSP slope in response to 30 min 10-kHz sine waveform, 80 V/m in radial direction. Error bars indicates SEM. *N*, number of slices. Blue (control), red (stimulation).

Whereas all the prior results used brief application of electric fields, we further tested whether stimulation for a longer period (i.e., 30 min) can induce lasting effects on fEPSP under the hypothesis that small effects could be amplified with longer stimulation duration. Stable baseline fEPSP was recorded every 30 s for over 20 min before stimulation and 60 min after stimulation. Electrical stimulation was done using sinusoidal 1- and 10-kHz stimulation with 80-V/m electric field intensity (Fig. 4) and effect on fEPSP was analyzed for condition (i.e., sham, stimulation) and time (i.e., immediately, 30 and 60 min after termination of stimulation). A repeated measure ANOVA revealed no significant effects for stimulation condition (1 kHz: *F*_(1,27)_ = 0.113, *p* = 0.739; 10 kHz: *F*_(1,23)_ = 0.09, *p* = 0.767), time (1 kHz: *F*_(2,54)_ = 0.024, *p* = 0.97; 10 kHz: *F*_(2,46)_ = 1.01, *p* = 0.375), and no interactions (1 kHz: *F*_(2,54)_ = 1.01, *p* = 0.37; 10 kHz: *F*_(2,46)_ = 1.92, *p* = 0.158).

### Bayesian analysis for supporting null hypothesis

Since these negative results may support either evidence of absence (provide support for null hypothesis) or absence of evidence because of lack of statistical power, we performed Bayes factor hypothesis testing for fEPSP evoked during 80 V/m stimulation applied in radial direction (parallel to somato-dendritic axis of pyramidal neurons) for 1- and 10-kHz sinusoidal, symmetric, and asymmetric waveforms. Moderate evidence was found for the absence of effect using 80 V/m, 10-kHz sinusoidal waveform, meaning that the observed data were ∼ 3× more likely to be under the null hypothesis than the alternative (BF_+0_ = 0.34 with median posterior δ= 0.187, 95% CI = [−0.177,0.560]), and anecdotal evidence for absence of effect in 1-kHz sinusoidal stimulation, meaning that the observed data were 1.67× more likely to be under the null hypothesis than the alternative (BF_+0_ = 0.63 with median posterior δ=−0.334, 95% CI = [−0.924,0.210]).

Using Bayes factor in symmetric pulse waveforms showed that data observed in 10 kHz is ∼ 3× more likely to be under the null hypothesis; providing moderate evidence for null (BF_+0_ = 0.33 with median posterior δ=−0.122, 95% CI = [−0.665,0.402]) whereas observed data in 1 kHz the data provided anecdotal evidence for null hypothesis: data were 1.33× more likely to be under the null hypothesis (BF_+0_ = 0.75 with median posterior δ= 0.369, 95% CI = [−0.162,0.943]). The data observed in during asymmetric pulse stimulation provided anecdotal evidence for both 1- and 10-kHz stimulation, meaning the observed data were 2.13× and 1.23× more likely to be under the null hypothesis, respectively (1 kHz: BF_+0_ = 0.47 with median posterior δ=−0.081, 95% CI = [−1.004,0.789], 10 kHz: BF_+0_ = 0.81 with median posterior δ=−0.42, 95% CI = [−0.216,1.143]).

Bayesian repeated measure ANOVA revealed strong evidence (1 kHz: BF = 0.1; 10 kHz: BF = 0.3) in support of the null hypothesis regarding effect of time (effect on EPSP immediate, 30 min, or 60 min after stimulation) and moderate evidence (1 kHz: BF = 0.4, 10 kHz: BF = 0.3) in support of the null hypothesis regarding effect of stimulation condition (i.e., sham vs stimulation on). Regarding interactions, Bayesian analysis revealed moderate and anecdotal evidence in support of the null hypothesis for 1 and 10 kHz, respectively (1 kHz: BF = 0.35, 10 kHz: BF = 0.8).

### Effect of kHz stimulation on hippocampal γ oscillations

**Figure 5.**
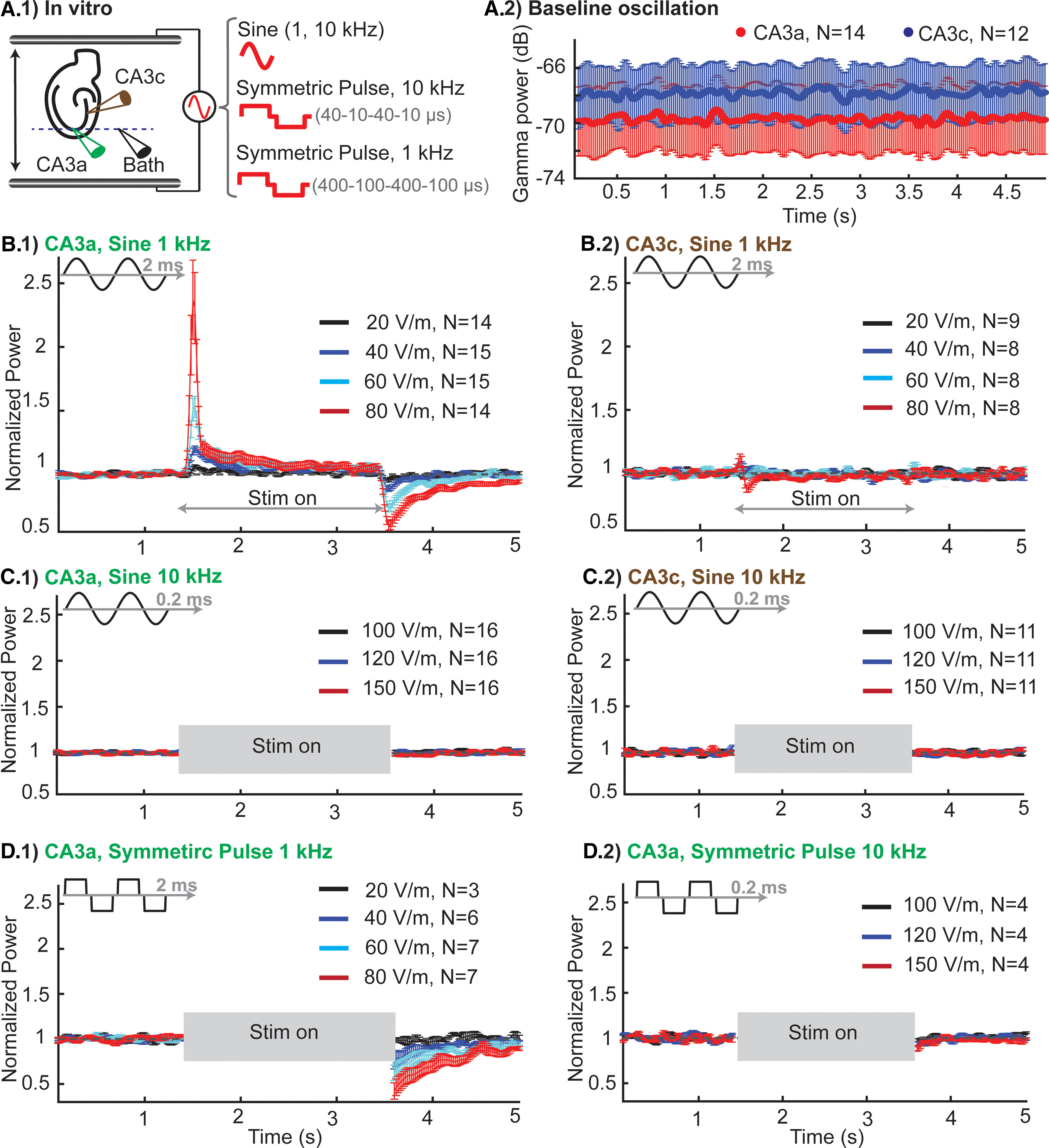
Sensitivity of hippocampal γ oscillations during application of 1- and 10-kHz sinusoidal and square waveform stimulation. ***A***, Rat in vitro model of γ oscillation. ***A.1***, experimental setup: spatially uniform electric field was applied across hippocampal slices in an interface chamber. Recording of γ oscillation from CA3a and CA3c relative to bath electrode to minimize stimulation noise. ***A.2***, Mean (±SEM) of baseline γ power (in dB) for CA3a and CA3c across slices. ***B***, Mean (±SEM) of normalized γ power across slices for 2 s of stimulation (between 1.5 and 3.5 s) using 1-kHz sinusoidal waveform with different field intensities recorded from CA3a (***B.1***) and CA3c (***B.2***). ***C***, Mean (±SEM) of normalized γ power across slices for 2 s of stimulation (between 1.5 and 3.5 s) using 10-kHz sinusoidal waveform with different field intensities recorded from CA3a (***C.1***) and CA3c (***C.2***). ***D***, Mean (±SEM) of normalized γ power across slices for 2 s of stimulation (between 1.5 and 3.5 s) using 1-kHz symmetric pulse waveform with different field intensities (***D.1***) and 10-kHz symmetric pulse waveform with different field intensities (***D.2***) recorded form CA3a region of rat hippocampus. *N*, number of slices.

Uniform unmodulated 1- and 10-kHz electric fields were applied across hippocampal slices exhibiting γ oscillations under carbachol perfusion (Fig. 5*A.1*). Oscillations were typically stable over ∼3 h, and experiments started after verifying stabilization of γ oscillation power. We evaluated the sensitivity of γ network activity to stimulation with kHz electric fields. Each stimulation was 2 s long and signals were recorded in frames of 5 s [acute effect, 5 s frame length (1.5 s pre, 2 s stim, 1.5 post), 80–100 frames per slice]. γ Oscillation was recorded from both CA3a and CA3c region of hippocampus. There was no significant difference in baseline γ power between the two recording locations (CA3a, *N* = 14; CA3c, *N* = 12, ns; Fig. 5*A.2*).

Consistent with previous reports (Reato et al., 2010; Esmaeilpour et al., 2020), low kHz stimulation generated transient effect at the onset of stimulation as well as a sustained effect in CA3a region (Fig. 5*B.1*). This muted sustained effect is presumably reflecting homeostatic network regulation to bring the network back toward equilibrium (e.g., baseline oscillatory level). Moreover, stimulation produced a poststimulation suppression of oscillation (Figure 5*B.1, C.1*), which is a marker of network rebound from homoeostatic adaptation (Reato et al., 2010). γ Oscillation recorded from CA3c region was not modulated during stimulation (Fig. 5*B.2*), highlighting the importance of electric field direction relative to somato-dendritic axis of pyramidal neurons for somatic polarization (Radman et al., 2009).

**Figure 6.**
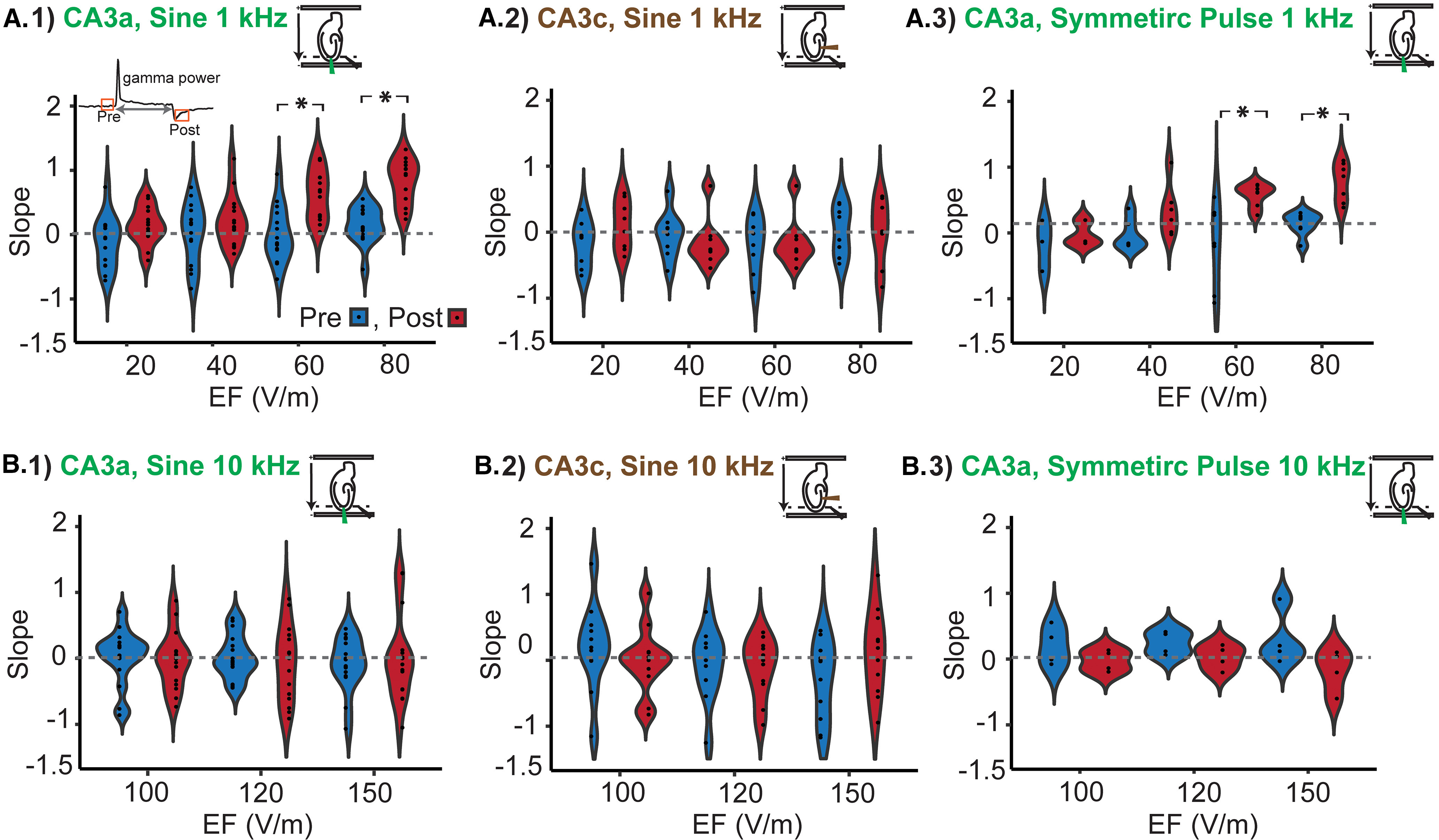
Post stimulation suppression of average γ oscillation power. ***A***, Slope of mean γ oscillation power (illustrated in Fig. 5) measured from 300-ms window immediately before and after 2 s of stimulation using 1-kHz sine waveform recorded from CA3a (***A.1***) and CA3c (***A.2***) and symmetric pulse waveform electrical stimulation recorded form CA3a region (***A.3***). ***B***, Slope of γ oscillation immediately before and after 10-kHz stimulation recorded from CA3a (***B.1***) and CA3c (***B.2***) using sinusoidal and symmetric pulse waveform recorded from CA3c region (***B.3***). Red, poststimulation γ slope. Blue, prestimulation γ slope. *p < 0.05.

Because of technical concerns of reliably removing stimulation artifact during 10-kHz sinusoidal stimulation and symmetric pulse waveforms, oscillation data were analyzed comparing only the prestimulation and poststimulation time windows (Fig. 5*C*,*D*). We defined slope of average γ power (see Materials and Methods) measured in 300-ms window immediately after termination of stimulation as a metric to quantify poststimulation suppression (Fig. 6).

Significant poststimulation suppression was detected using 1-kHz sinusoidal waveform with field intensities ≥60 V/m in CA3a region (γ power slop: 60 V/m, post: 0.62 ± 0.010, pre: 8.5 × 10^−4^ ± 0.11, *N* = 15, *p* < 0.001; 80 V/m; post: 0.83 ± 0.09, pre: 0.15 ± 0.074, *N* = 14, *p* < 0.001; Fig. 6*A.1*); however, in CA3c region, no change was detected in slope of γ power immediately after stimulation (Fig. 6*A.2*). Similarly, symmetric pulse 1-kHz stimulation using intensities ≥ 60 V/m induced significant rebound after stimulation (γ power slope: 60 V/m, post: 0.58 ± 0.06, pre: −0.15 ± 0.23, *N* = 7, *p* < 0.01; 80 V/m, post: 0.77 ± 0.11, pre: 0.13 ± 0.64, *N* = 7, *p* < 0.01; Fig. 6*A.3*). Increasing stimulation frequency from 1 to 10 kHz abolished the effect. No effect was observed in 10-kHz symmetric pulse and sinusoidal stimulation using poststimulation suppressions as an index even when testing still higher electric field strength (i.e., 100, 120, and 150 V/m; Fig. 6*B*).

## Discussion

There is a long-standing interest in explaining neuronal responses to kHz range electrical stimulation (Katz, 1939; Ward, 2009) with many results still inconclusive or without satisfactory theoretical treatment. Various forms of kHz neuromodulation techniques have shown promise in managing chronic pain (Al‐Kaisy et al., 2015; Thomson et al., 2018) improving motor function in Parkinson’s disease (Harmsen et al., 2019) and modulating excitability of human motor cortex (Terney et al., 2008; Chaieb et al., 2011; Antal and Paulus, 2013). Variations of kHz stimulation (electrode position, pulsed/sinusoidal waveforms) has been characterized in a broad range of applications including physiotherapy (Ward, 2009; Medeiros et al., 2017), ceasing abnormal neuronal activity (Kilgore and Bhadra, 2014; Lempka et al., 2015; Pelot and Grill, 2020) or generating spontaneous or asynchronous firing (Rubinstein et al., 1999; Litvak et al., 2003; Crosby et al., 2017). In contrast, it is a fundamental property of cells that the parallel leak conductance and capacitance of outer membrane forms an equivalent of a filter that attenuates neuronal responses to inputs with high-frequency components. This intrinsic low pass filtering property of neuronal membrane explains various electrophysiological finding at the cellular and neuronal network level on limited sensitivity to kHz electric fields (Deans et al., 2007; Reato et al., 2010), although once polarized, ions channel have some kinetics with sub-ms time constants (Zhang et al., 2006; Zhao et al., 2014). At the same time, some application using AM kHz stimulations are based on the assumption neurons are insensitive to the unmodulated kHz component (Goats, 1990; Ward, 2009; Grossman et al., 2017). We therefore set out to clarify the sensitivity of the brain to unmodulated, uniform, 1- or 10-kHz sinusoidal (e.g., single frequency band) fields between 1 and 150 V/m.

The acute brain slice model has been extensively used as a model system to screen for effects of a broad range of stimulation waveform and intensities, including subthreshold fields (Bikson et al., 2004; Rahman et al., 2013, 2017; Jackson et al., 2016) and is generally among the most characterized experimental system in neuroscience (Ranieri et al., 2012). Consistent with screening for a broad range of possible effects, single and paired fEPSPs are sensitive to changes either in presynaptic or postsynaptic excitability. Oscillations are similarly highly sensitive to changes in excitatory and inhibitory cellular function through mechanism of amplification specific to network’s architecture and level of activity (Reato et al., 2010, 2013; Jackson et al., 2016). Furthermore, field measures are insensitive to intracellular artifacts specific to kHz fields (Lesperance et al., 2018; FallahRad et al., 2019). A change in fEPSP or oscillations in response to kHz electric fields are thus robust and broad indicators of changes in brain function, which, if positive, can then be followed by more specific testing to identify cellular targets.

We systematically evaluated responses to a range of waveforms (sinusoidal, symmetric, asymmetric pulses), intensities, 1- and 10-kHz frequencies, electric field direction (radial, tangential), stimulation duration (30 s typical, 30 min), and during and PF effects. While impractical to test all combinations, our overall experimental strategy was intended to identify responses. We focused (number of slices) on 80 V/m but tested a range of intensities in case responses are not monotonic. Given established sensitivity to DC fields of slice prep neurons (Bikson et al., 2004; Jackson et al., 2016), we conducted within-slice positive controls for general sensitivity to electric fields. By any measure, fEPSPs were not modulated by kHz waveform tested, regardless of intensity (up to 80 V/m), waveform, direction, or timing; 1- but not 10-kHz electric field modulated ongoing network oscillations. The intensity required for 1-kHz electric fields to modulate γ oscillation was substantially higher than for low-frequency (e.g., ∼100 Hz) fields (Esmaeilpour et al., 2020). This overall lack of sensitivity is consistent with prior kHz-stimulation mechanistic studies (Couto and Grill, 2016; Lempka et al., 2015; Negahbani et al., 2018; Esmaeilpour et al., 2020) and the established low-pass filtering characteristics of neuronal membranes to electrical stimulation (Deans et al., 2007; Reato et al., 2013).

Our results are limited by several factors. It is never possible to exclude β errors, though our use of a high SNR experimental system, with multiple slices and numerous repetitions per condition per slice, as well as within slice positive DC controls, together suggest such undetected effects would be variable or small in any case. Alternative mechanisms of electric fields such as ion concentration changes (Bikson et al., 2001; Shapiro et al., 2020; Wang et al., 2020), fiber block (Zhang et al., 2006; Zhao et al., 2014; Patel and Butera, 2015; Shapiro et al., 2020) and transverse axonal polarization (Wang et al., 2018) are suggested for kHz stimulation at very high intensities. However these very high intensities are not expected in existing clinical applications, such as SCS, with targeted tissue some mm away from the electrode (Lempka et al., 2015; Idlett et al., 2019). As emphasized throughout this article, these results are limited by any biophysical features absent from our experimental model system. Effective kHz stimulation with intensities comparable to these clinical applications would require a transduction mechanism with an especially fast time constant that is absent in acute rodent brain slice.

Following the quasi-uniform assumption (Bikson et al., 2013a, 2015; Khadka et al., 2019), we applied uniform fields, leaving open the possibility that geometry-sensitive effects were missed (Idlett et al., 2019). Our results are limited to the intensities and specific waveforms tested, though a range of pulse-shapes were considered. We cannot consider possible mechanisms not captured by the hippocampal brain slice, such as a highly sensitive subtype of neurons (Rubinstein et al., 1999; Litvak et al., 2003; Lee et al., 2020), vascular responses (Cancel et al., 2018), or temperature (Zannou et al., 2019a,b); the latter in fact increases with kHz frequency.

## Synthesis

Reviewing Editor: Upinder S. Bhalla, National Centre for Biological Sciences, Tata Institute of Fundamental Research; Decisions are customarily a result of the Reviewing Editor and the peer reviewers coming together and discussing their recommendations until a consensus is reached. When revisions are invited, a fact-based synthesis statement explaining their decision and outlining what is needed to prepare a revision will be listed below. The following reviewer(s) agreed to reveal their identity: NONE. Note: If this manuscript was transferred from JNeurosci and a decision was made to accept the manuscript without peer review, a brief statement to this effect will instead be what is listed below.

The reviewers and I felt that this was a sound paper clearly presenting a

negative result of value to the field. This is useful because of the wide

use of a range of brain stimulus protocols including in this frequency range,

and points to the need for better understanding of the mechanisms of such

stimuli. There were a few small changes suggested to improve the paper.

1. The authors should clarify several figures, including slope for fEPSP/gamma,

duration of kHz stimulus. There is scope for improving the presentation of

data points using violin plots, and other points brought up by the reviewers.

2. The reviewers suggest several discussion points that would improve the

paper. These include a better assessment of the statement about low-pass

filtering, placing the surprising findings in broader context specially with

regard to changes in ion concentrations, and considering the magnitude of the

kHz electric field.

In addition the reviewers have a number of other suggestions that would

help the presentation.

Reviewer 1:

This paper provide negative results showing that kHz stimulation has no effect on field potential recording of EPSP and gamma oscillation activity in hippocampal slice of rat. Although it is negative result, it is still a much needed study to understand the effect of kHz stimulation on nerve/neuron activity. Currently kHz stimulation is successfully used in clinical applications, but it mechanisms of action are poorly understood.

This study shows that the field potential recordings of EPSP and gamma oscillation activity in hippocampal slice of rats are not influenced by kHz stimulation. Although this is a negative result, it is still very important for understanding the effect of kHz stimulation on nerve/neuron. Although the clinical application of kHz stimulation is very successful, the mechanism of action is poorly understood.

1. Please define the slope for the fEPSP and gamma activity. How these slopes are measured? Different slope are showing in different figures as the major results, but the definition of these slopes and how they were measured are not described.

2. Please indicate in each figure what is the duration of the kHz stimulation. 2 seconds? 30 seconds? Also, in the result section, the duration of kHz stimulation should be clearly indicated for each data/result.

3. How is the 80V/m maximal intensity selected or determined? Why not go higher?

4.Fig.1 shows data are collected during 30 min kHz stimulation, but data only presented before and after the 30 min stimulation in Fig.4.

5. Discussion: “This intrinsic low pass filtering property of neuronal membrane explains various electrophysiological finding at the cellular and neuronal network level on lack of sensitivity to kHz electric fields ...” The neural membrane can be alternatively depolarized and hyperpolarized by 10 kHz biphasic stimulation and the K channel can follow up to 5 khz and Na channel can follow up to 15 kHz (J Comput Neurosci. 2014 Oct;37(2):377-86. and IEEE Trans Biomed Eng. 2006 Dec;53(12 Pt 1):2445-54.). So, it is not appropriate to say that neural membrane is a low pass filter.

6. It is a surprise that the results in this paper is negative. Recent studies have shown that post-stimulation block can be induced by prolonged kHz stimulation, indicating a possible mechanism that long-duration kHz stimulations can significantly change the K and Na ion concentrations thereby blocking nerve conduction (Neuromodulation. 2020 Aug;23(6):747-753. and Neuromodulation. 2020 Aug 6. doi: 10.1111/ner.13241. Online ahead of print). It should be discussed why the possible changes in ion concentrations have no effect on your results.

Reviewer 2

This manuscript solidly demonstrated that kHz constant-magnitude neuromodulation on a brain slice is not effective to make notable electrophysiological changes. This raises the question of how the published in vivo effects of kHz neuromodulation can be mechanistically explained.

Overall, this is a thorough negative result paper on kHz neuromodulation (in slice). The results and interpretation are summarized well in the abstract. It can be published as is, though Figure presentation of quasi-box plots and data points can be improved perhaps by widening each box or using violin plots instead. However, this is up to the authors. The discussion is fair and succinct.

Line 47: Delete “On the other hand,"

Kuster et al. (2017) in the reference list, but not cited.

Line 103; Is the ketamine dosage correct for deep anesthesia?

Lines107/113 D- or d-glucose

Line 217: field/filled

Text in Fig 1: repetitions (?)

Fig 5 caption: perhaps it is better to state “normalized gamma power”.

Line 389: treatment. (delete the space); also, has showed/have shown

What is the magnitude of the kHz electric field recorded by the electrode during stimulation periods? The question is if the field caused by el. stimulation is dampened in a frequency-dependent manner similar to the transmembrane effect.

## Author Response

Response to reviewers:

Synthesis of Reviews:

The reviewers and I felt that this was a sound paper clearly presenting a negative result of value to the field. This is useful because of the wide use of a range of brain stimulus protocols including in this frequency range, and the study points to the need for better understanding of the mechanisms of such stimuli. There were a few small changes suggested to improve the paper.

We appreciate the editor and reviewers detailed reading and constructive suggestions.

1. The authors should clarify several figures, including slope for fEPSP/gamma, duration of kHz stimulus. There is scope for improving the presentation of data points using violin plots, and other points brought up by the reviewers.

We agree. All the required information is added, and figures are updated using violin plots.

2. The reviewers suggest several discussion points that would improve the paper. These include a better assessment of the statement about low-pass filtering, placing the surprising findings in broader context specially with regard to changes in ion concentrations, and considering the magnitude of the kHz electric field.

Thank you for the constructive comment. Discussion section is updated accordingly.

In addition, the reviewers have a number of other suggestions that would help the presentation.

Reviewer #1:

This paper provide negative results showing that kHz stimulation has no effect on field potential recording of EPSP and gamma oscillation activity in hippocampal slice of rat. Although it is negative result, it is still a much needed study to understand the effect of kHz stimulation on nerve/neuron activity. Currently kHz stimulation is successfully used in clinical applications, but it mechanisms of action are poorly understood.

This study shows that the field potential recordings of EPSP and gamma oscillation activity in hippocampal slice of rats are not influenced by kHz stimulation. Although this is a negative result, it is still very important for understanding the effect of kHz stimulation on nerve/neuron. Although the clinical application of kHz stimulation is very successful, the mechanism of action is poorly understood.

Thank you, we added a Bayesian statistics section to further discuss the negative results.

1. Please define the slope for the fEPSP and gamma activity. How these slopes are measured? Different slope are showing in different figures as the major results, but the definition of these slopes and how they were measured are not described.

The methods section is updated to include detailed information about slope measurement in both fEPSP and gamma oscillation.

2. Please indicate in each figure what is the duration of the kHz stimulation. 2 seconds? 30 seconds? Also, in the result section, the duration of kHz stimulation should be clearly indicated for each data/result.

Thank you, the text is updated accordingly.

3. How is the 80V/m maximal intensity selected or determined? Why not go higher?

The paper clarifies, in some experiments fields up to 150 V/m were tested which covers the range expected by some clinical modalities testing kHz stimulation (e.g. SCS) and well exceeds intensities expected in others (e.g. interferential stimulation), but is below intensities shown in other cited experimental papers to be effective for kHz stimulation.

4.Fig.1 shows data are collected during 30 min kHz stimulation, but data only presented before and after the 30 min stimulation in Fig.4.

Our goal in that section was to study the effect after long duration of stimulation. For technical limitations, the sampling frequency was reduced in these longer-term experiments such that stimulation artifact could not be reliably removed from fEPSP during stimulation. We have clarified it in method section.

5. Discussion: “This intrinsic low pass filtering property of neuronal membrane explains various electrophysiological finding at the cellular and neuronal network level on lack of sensitivity to kHz electric fields ...” The neural membrane can be alternatively depolarized and hyperpolarized by 10 kHz biphasic stimulation and the K channel can follow up to 5 khz and Na channel can follow up to 15 kHz (J Comput Neurosci. 2014 Oct;37(2):377-86. and IEEE Trans Biomed Eng. 2006 Dec;53(12 Pt 1):2445-54.). So, it is not appropriate to say that neural membrane is a low pass filter.

Yes. The statement has been clarified as suggested. We also expanded our discussion to emphasize our results are applicable to the target, waveforms and intensities tested.

6. It is a surprise that the results in this paper is negative. Recent studies have shown that post-stimulation block can be induced by prolonged kHz stimulation, indicating a possible mechanism that long-duration kHz stimulations can significantly change the K and Na ion concentrations thereby blocking nerve conduction (Neuromodulation. 2020 Aug;23(6):747-753. And Neuromodulation. 2020 Aug 6. doi: 10.1111/ner.13241. Online ahead of print). It should be discussed why the possible changes in ion concentrations have no effect on your results.

We expanded our discussion section to discuss this important issue and add additional statistics.

Reviewer #2

This manuscript solidly demonstrated that kHz constant-magnitude neuromodulation on a brain slice is not effective to make notable electrophysiological changes. This raises the question of how the published in vivo effects of kHz neuromodulation can be mechanistically explained. Overall, this is a thorough negative result paper on kHz neuromodulation (in slice). The results and interpretation are summarized well in the abstract. Figure presentation of quasi-box plots and data points can be improved perhaps by widening each box or using violin plots instead.

The discussion is fair and succinct.

Thank you for the positive feedback. The boxplots are replaced with violin plots to represent data distribution better.

Some specific points:

Line 47: Delete “On the other hand,” Corrected.

Kuster et al. (2017) in the reference list, but not cited. Corrected.

Line 103; Is the ketamine dosage correct for deep anesthesia? Yes, it is correct.

Lines107/113 D- or d-glucose. Its D-glucose, corrected.

Line 217: field/filled Corrected.

Text in Fig 1: repetitions (?). Corrected.

Fig 5 caption: perhaps it is better to state “normalized gamma power”. Corrected.

Line 389: treatment. (delete the space); also, has showed/have shown. Corrected.

What is the magnitude of the kHz electric field recorded by the electrode during stimulation periods? The question is if the field caused by el. stimulation is dampened in a frequency-dependent manner similar to the transmembrane effect.

Electric field was calibrated before each experiment and during experiment both electrodes are place in iso-potential line to remove artifact and cannot represent the electric field across neuronal membrane in somato-dendritic axis.
